# Tales of precarity: A reflexive essay on experiencing the COVID pandemic as a social work educator on a precarious contract

**DOI:** 10.1177/1473325020973311

**Published:** 2021-03

**Authors:** Joe Whelan

**Affiliations:** School of Applied Social Studies, University College Cork, County Cork, Ireland

**Keywords:** Precarity, social work, COVID-19, Corona Virus, lived experiences, Ireland

## Abstract

This is a reflexive essay that documents my experience of being a social work educator and early career researcher on a precarious contract in a university in the Republic of Ireland during the COVID-19 global pandemic. At the time of writing and during the different phases of the pandemic that have come to pass so far, I have been employed as a coordinator and lecturer on a Bachelor of Social Work (BSW) programme, while also teaching on and contributing to a number of other programmes at both undergraduate and postgraduate level. Like educators the world over, I was thrust into a space where teaching, overnight, migrated online. Like many others also, I experience this as someone with little job security, who has moved from one contract to another and who has no certainty as to what the future will bring in respect to ongoing employment. At the same time, I am keenly conscious that our students, from our first years who just started out to our fourth years on the cusp of graduation, are also reckoning with enhanced precarity and uncertainty. I aim to unpack the implications of this over the course of this essay by drawing on my recent lived experiences. In respect to method, I began documenting my experiences from the day the university closed through contemporaneous notetaking in a research journal. I also began to archive central university correspondence relating to the pandemic. I draw on these notes and materials here. While the purpose here is to offer a narrative that is not over-theorised, I do attempt to link my experiences to wider social, cultural, and political understandings so that what is presented can be described as autoethnography.

## Context

There is little need to present a detailed context here outside of saying that, at the time of writing, I am a qualified social worker with a PhD who teaches in a university in the Republic of Ireland and have done so for several years. I teach mainly on an accredited, four-year, BSW, undergraduate, degree programme which each year qualifies up to twenty-five social workers. As well as teaching in-class, I supervise student research and I tutor students on placement.

Outside of this, the precarity is the context. Like so many others that work in higher education, I am employed via a short-term contract. I have virtually zero job-security outside of the terms of that contract which may or may not be renewed depending on the needs of the university at the time of its lapse. I undoubtedly appear to other, even more precarious colleagues, to possess the trappings of security, an office, a staff profile page, yet, in reality, I am ultimately expendable. I am also paid far below that of more established colleagues, many of whom have had to thread the very same waters I now thread.

It is not lost on me that I also teach within a discipline that professes to value social justice yet is itself ensconced within a university model that arguably eschews the values of social justice and presents education as a commodity. Of course, my situation is far from unique and the general trend of which it is a part undoubtedly mirrors the situation seen in universities in the United Kingdom (UK) where recent reports have suggested that ‘casualisation’ is endemic (University and College Union, 2019).

This covers the context of my personal employment situation at the time of writing. In respect to contextualising the measures taken in the wake of the pandemic, the university closed its doors to students and almost all staff alike on the 12^th^ of March 2020. It is from this point on that the reflections presented here are drawn. Utlising an autoenographic approach (Ellis, 2004), I draw on data in the form of excerpts of my observations recorded in a research journal. For clarity, I label each excerpt by the week it was made and the numerical order it was made in. So, for example, my third entry in week one would be labelled as follows: (Week 1, entry 3).

## In these strange and uncertain times…

‘In these strange and uncertain times’ is a phrase we now remark daily in how we communicate and one I immediately found myself using variants of when corresponding with colleagues and students alike. I noted this in my journal in the following terms:

It’s strange how emails which, in the past, only hoped to ‘find you well’ now hope to ‘find you well in these strange and uncertain times’ (Week 2, entry 6).

This is not a glib observation, rather I wish to suggest that this specific phraseology, which has emerged in response to the overt levels of strangeness and uncertainty that the pandemic has wrought, serves as an tacit acknowledgement that we, as human beings, generally dislike uncertainty and wish ourselves and other people to be free of it in the first instance and to at least be well in the face of it, if not quite free.

Yet, there is an irony in this acknowledgement of uncertainty too. For the precariously employed researcher or academic, uncertainty is part and parcel of existence and has merely been exacerbated by the pandemic. My personal uncertainty has been grinding, burrowing inward, tempering and infecting all my experiences, my small triumphs, my bigger successes and my failures too. This is because precarity itself feels like failure. I feel I have failed by still being precarious. Sometimes this takes the form of feeling undervalued, on other occasions it is simply a case of feeling that I must not be ‘good enough’ to warrant security. My precarity, therefore, is something I am always aware of, yet, it did begin to become more pronounced and raise new questions in the wake of the COVID-19. Though written in a very different context, I am nevertheless inspired here by Morris (2018) and her work with the concept of ‘haunted futures’. Reflecting this, in an entry made in my research journal in the first week of the university closure, I ask:

What is going to happen to me now? How will the university deal with precarious staff? My contract is ending soon, will it be renewed or left to lapse? What will I do in the case of the latter? (Week 1, entry 4).

As I began to reflect upon and unpack this, I also began to feel there was a clear double-standard on the part of my employer that was perhaps always present, but which was now easier to spot in the face of global uncertainty. To evidence this, at least anecdotally, I submit that in the first six days of the closure of my own university, *at least* six emails that were centrally communicated to all staff mentioned the word *uncertainty* in an overtly pejorative sense. Below is just one example of how this was often worded:

Dear Staff & Students,

We appreciate that this is a time of *anxiety* and *uncertainty* for our community…

Many other emails made mention of the ‘unprecedented’ or of the ‘challenging’ circumstances the university now faced. Others still addressed, directly, the mental health implications that such uncertainty might wreak, counselling self-care. Alongside this, were the rousing communications made from on high. These congratulated the efforts made by those on the ‘front-lines’ while attempting to stir souls and ask for yet more effort in the ‘battles’ to come.

There is a lot going on in this mix, much of it undoubtedly genuine and heartfelt. Yet it also reeks of hypocrisy and double-standard on the part of the university as an employer, a hypocrisy that, on the one hand, counsels against the negative effects of uncertainty and, on the other, has maintained employment practices that actively promote uncertainty and force people to live precariously. Documenting my response to this growing sense of hypocrisy, this made me feel, angry, sad and often frustrated and this is reflected in the entry below:

If I get one more email telling me to mind myself or professing to give a shit about my mental health in these ‘uncertain’ times, I might actually crack-up. Piss off. If you want me to feel certain and secure give me decent pay and conditions not an image of a fucking waterfall (Week 4, entry 4).

Reflecting further, there is a question to be asked: now that universities have acknowledged the negative impact of uncertainty upon persons, how will they address this in their employment practices? As my contract dwindles, I also feel hopeless at times. I both see, and am personally caught up in, a horrible irony that, on the one hand, has seen precarious staff on casual contracts used to generate surpluses for universities thus offsetting potential future risks, yet, now that universities are facing unprecedented risk, this is being transferred to precarious staff losing jobs so universities don’t use up those same reserves.

It is worth noting that for me, the feelings associated with precarity described here generally have the character of a background hum, much like that of someone cutting grass in the aural distance, you hear it and are aware of it, yet it is not immediate. What *was* immediate, however, was concern for my students and for how I would see out the remaining semester of teaching. I recorded this in the following terms:

Jesus Christ, I would hate to be a student right now. The stress! (Week 2, entry 3).

## WiFi connection strong…but who is connected?

While my own personal employment precarity became exacerbated by COVID-19 and I now faced contract renewal with more trepidation than usual, my teaching also needed to undergo a drastic change meaning I would be affected in how I delivered my teaching and my students would be affected in how they received materials. In the case of my particular university, no break was taken before teaching resumed. Like all universities, we closed on Thursday 12^th^ of March 2020. From early that day I knew by rumour that the university would close. I remember standing in the office of a colleague, each of us refreshing screens as we awaited the official word to come. Realising that I was living through something historic, it was then that I decided to document events in a research journal. I made the first entry in my own office later that day and after official word had finally come. It reads:

We have just gotten word by email that we are closing. I wonder when I will see this place again, I wonder if I will ever see it again. I have a sense of finality about leaving for some reason but also resolve, resolve to do what, I’m not really sure (Week 1, entry 1).

Without skipping a beat, teaching resumed in an online capacity immediately. The very next day, I was prepping online materials at home for the following week. I would have four modules to see out online as well as ongoing communication with supervisees and tutees. I would do this as someone parenting alone with three children.

In ordinary circumstances, my classroom teaching is underpinned by the social construction of knowledge. This devolves upon the belief that by encouraging students to actively engage through things like class discussion and small group work they will come to knowledge in a much more active way and useful way. Perkins (1999: 08), in presenting a philosophical argument for social constructionism as a teaching approach, suggests that:

The stimuli that we encounter, including messages from others, are never logically sufficient to convey meaning. To some extent the individual always has to construct or reconstruct what things mean. It thus makes sense to organise learning to reflect this reality.

Yet, how to capture this online and how would the students respond in a climate of unsureness, challenge and uncertainty? After careful consideration of what I felt both I and my students could manage, I decided to focus on generating discussion in an online forum. I supplied students with readings and lecture notes and opened corresponding discussion boards with prompts, inviting students to interact with their peers while clarifying that those who could not do so, would not be penalised in any way.

Immediately, I saw that many students were struggling with this. For some, it was limited access to broadband, for others it was just not feasible to spend time engaging in reading and discussion in the face of increased caring responsibilities; schools had closed, many of our students are parents. For others still, it was the very genuine worry, uncertainty and anxiety that the circumstances evoked. This caused me to reflect on the various types of uncertainty our students were facing:

A lot of things that were in play for our students earlier this year have now been thrown into flux. Students have had placements cut short and remain unsure of if and when these can safely resume. Students who are finishing and qualifying this year, who were perhaps hoping to begin their careers as social workers, are now facing a very uncertain jobs market. All this and the very real fear of the virus. It’s tough time to be a student (Week 3, entry 2).

Reflecting on this further, it struck me that the word ‘uncertainty’ was again replete here. Our students faced so much uncertainty and trepidation that, in many ways, continuing to teach and to expect them to engage during a global pandemic felt, at times, ridiculous. Yet I and others would continue do so.

I also found myself reflecting on what this teaching was like and how on how it was being experienced. I couldn’t help but feel that something was lost in online delivery, something irreplaceable and something so important to social work education:

There is a materiality to teaching in a classroom, a presence that cannot be replaced in an online forum. When we work together in a classroom, we pool our common humanity for a brief time. Teaching, meeting, talking online does not seem to capture this presence, and being present, being connected is so, so important to social work (Week 4, entry 7).

There was a sadness to these thoughts and also a longing. Sadness that students faced such uncertainty, something I too felt and could empathise with, sadness that it might be a long time before the simple act of being present was possible again. Longing too, for the simplicity of that presence. All tempered by the sense that even when presence becomes possible, I may not be back to experience it:

I got the dreaded email form HR today. Your contract is due to expire….thank you for your services. Bastards (Week 7, entry 11).

## Closing thoughts

In this brief reflexive essay, I have drawn on the entries made in a research journal that aimed to capture my experiences as a precariously employed social work educator in the Republic of Ireland. The aim has been to capture and present my own sense of uncertainty and precarity and to elucidate what this has been like during the COVID-19 crisis. I also wanted to transmit a sense of how students face similar precarity and uncertainty. In doing so I encountered themes of uncertainty, sadness, anger and anxiety within my own subjective experiences and within my interpretation of student experiences. Without wishing to generalise from myself to others, it is probable that my personal experiences and the insight I have into my student’s experiences are not uncommon and are likely to be found in a range of contexts. Uncertainly is a part of life. Yet, I wanted to show here how it can be a deleterious and grinding aspect of experience. The circumstances thrust upon us all by COVID-19 have exposed an intense dislike of uncertainty, the word uncertainty itself being continuously used in an overtly pejorative sense. Yet those of us who experience precarious employment carry uncertainty with us on a continuous basis and the uncertainly of studentship is compounded by the virus.

I have also documented the immediate change to the nature of teaching that occurred as a result of COVID-19. As well as being something that had immediate ramifications, the shift to online teaching is something that will affect education into the future. At the time of writing, the prospect of a ‘blended’ learning environment seems very real and likely to be with us for the foreseeable future. For me, this exacerbates a sense of longing for the presence and human connection that in-class teaching quietly exemplifies. For social work and for the social professions in general, this connection feels vitally important. I therefore, worry that social work education, an education that is often simply about showing our students how to be with and alongside people, will suffer in the absence of opportunity to share our common humanity in some proximity to one another.
